# One Health Lens on Rabies: Human–Bat Interactions and Genomic Insights of Rabies Virus in Rural Lilongwe, Malawi

**DOI:** 10.3390/tropicalmed10040095

**Published:** 2025-04-04

**Authors:** Nathan Singano, Henson Kainga, Elisha Chatanga, Joseph Nkhoma, Gilson Njunga, Julius Chulu, Rabecca Tembo, Hirofumi Sawa, Walter Muleya

**Affiliations:** 1 Department of Biomedical Sciences, School of Veterinary Medicine, The University of Zambia, Lusaka P.O. Box 32379, Zambia; singanonathan@gmail.com; 2 Department of Veterinary Epidemiology and Public Health, Faculty of Veterinary Medicine, Lilongwe University of Agriculture and Natural Resources, Lilongwe P.O. Box 219, Malawi; hensonkainga@luanar.ac.mw; 3 Department of Veterinary Pathobiology, Faculty of Veterinary Medicine, Lilongwe University of Agriculture and Natural Resources, Lilongwe P.O. Box 219, Malawi; echatanga@luanar.ac.mw; 4 Central Veterinary Laboratory (CVL), Lilongwe P.O. Box 527, Malawi; joemnkhoma@gmail.com; 5 Department of Animal Health and Livestock Development, Lilongwe P.O. Box 2096, Malawi; gnjunga@tappmalawi.org (G.N.); juliuschulu09@gmail.com (J.C.); 6 Trustees of Agricultural Promotion Programme, P/Bag A21, Lilongwe P.O. Box 2096, Malawi; 7 Department of Pathology and Microbiology, School of Medicine, The University of Zambia, Lusaka P.O. Box 50110, Zambia; rabecca.tembo@unza.zm; 8 International Institute for Zoonosis Control, Hokkaido University, Sapporo 001-0020, Japan; h-sawa@ivred.hokudai.ac.jp; 9 Institute for Vaccine Research and Development, Hokkaido University, Sapporo 001-0021, Japan

**Keywords:** rabies virus, knowledge, practice, whole-genome sequencing, phylogenetic analysis, human–bat interaction, risk factors, Malawi

## Abstract

Rabies, a fatal zoonotic disease, affects humans, domestic animals, and wildlife predominantly in Africa, Asia, and Latin America. In Malawi, rabies virus (RABV) is primarily transmitted by infected dogs, impacting humans and cattle. Lyssavirus has also been documented in insectivorous bats. A community survey near bat roosts assessed knowledge, attitudes, and practices regarding bat-borne zoonoses. Bat samples were tested for lyssavirus using RT-PCR, and RABV genomes from human and domestic animals were sequenced and analysed phylogenetically. The survey revealed that 50% of participants consumed bat meat, and 47% reported bats entering their homes. Reduced bat presence indoors significantly lowered contact risk (aOR: 0.075, *p* = 0.021). All 23 bat samples tested negative for lyssavirus. Malawian RABV genomes, 11,801 nucleotides long, belonged to the Africa 1b lineage, showing >95% similarity with GenBank sequences. Phylogenetic analysis indicated close clustering with strains from Tanzania, Zimbabwe, and South Africa. Human and cattle strains shared 99% and 92% amino acid similarity with dog strains, respectively, with conserved critical sites and unique substitutions across all five RABV genes. Frequent human–bat interactions pose zoonotic risks. While no lyssavirus was detected in bats, ongoing surveillance is crucial. This first comprehensive genome analysis of Malawian RABVs highlights their regional transmission and signifies the need for regional collaboration in rabies control, community education, and further study of genetic adaptations.

## 1. Introduction

Rabies is a fatal zoonotic viral disease affecting humans, domestic animals, and wildlife predominantly in Africa, Asia, and Latin America. The classical rabies virus (RABV), belonging to the genus Lyssavirus, is the causative agent of rabies [1]. The RABV genome is a single-stranded negative-sense RNA that encodes five structural proteins in this sequence: nucleoprotein (N), phosphoprotein (P), matrix protein (M), glycoprotein (G), and RNA polymerase (L) [2]. The nucleoprotein is responsible for the RNA encapsulation and transcription [3]. The phosphoprotein binds to the N-RNA template and aids in the transportation of viral nucleocapsids through interaction with the cytoplasmic dynein chain [4]. The matrix protein downregulates viral transcription and contributes to cytopathogenesis [5]. The glycoprotein is key for viral attachment to host cells and pathogenicity [6]. The L protein is a polymerase in the nucleoprotein core, essential to RNA transcription and replication [2]. RABV is transmitted through bites or scratches from an infected animal, particularly rabid dogs [7]. Rabies is responsible for approximately 59,000 global deaths annually, with Africa and Asia accounting for more than 95% of the cases [8].

Some species within the orders Carnivora (jackals) and Chiroptera (bats) are acknowledged as reservoirs of lyssavirus [9]. Beyond RABV, a spectrum of rabies-related lyssaviruses (RRVs) have been identified in *Miniopteridae* and other bat species [10]. These include, among others, the Lagos bat virus in Nigeria, the Shimoni bat virus in Kenya, and the Duvenhage virus in South Africa [11]. RABV in Africa is categorised into lineages 1a, 1b, 2, 3, and 4 [12], distributed across Northern Africa, Eastern and Southern Africa, Western and Central Africa, Southern Africa, and Egypt, respectively [13]. According to Kainga et al. [14], the RABV strains in Malawi belong to the Africa 1b lineage; however, this conclusion was based only on individual N and G genes [14].

Rabies virus in Malawi is primarily transmitted by infected dogs (*Canis familiaris*) through bites, infecting other hosts, including humans (*Homo sapiens*) and cattle (*Bos indicus*). The burden of human rabies in Malawi is estimated at 500 deaths per year [8], and children are the most affected [15]. From 2008 to 2021, 683 non-human confirmed rabies cases were reported in Malawi, with the highest being domestic dogs (*n* = 435), followed by cattle (*n* = 94). RABV has also been documented in insectivorous bats in Malawi [14]. Like many countries, livestock in rural Malawi coexist closely with people, and household structures often allow entry to domestic and wild animals for food and shelter [16]. Within these communities, bat meat is considered a culinary delicacy, and bat hunting is practised to a lesser extent, suggesting a lack of knowledge behind these human–bat interactions. Additionally, bat roosts have been observed in peri-urban areas of Lilongwe, the capital city [17]. Together, these observations highlight the shrinking gap between humans, domestic animals, and wildlife, thus increasing the risk of exposure to RABV.

These examples emphasise the significance of a One Health approach to understanding human, animal, and environmental health [18]. One Health surveillance is vital for investigating how human–bat interactions can result in the spillover of RABV and other lyssaviruses from animals to human populations at the human–wildlife interface [19,20]. Integrating genomic and epidemiological data within the One Health framework strengthens rabies surveillance by providing insights into the strains of RABV circulating between animals and humans. This study provides baseline epidemiological data for the development of coordinated multisectoral control strategies across human and animal populations. Research efforts in Malawi have predominantly focused on the health of humans and livestock, with minimal attention given to wildlife, specifically bats. Previous genomic studies on RABV in Malawi have focused on individual genes [14], but no full-length viral genome analysis has been conducted to date. Thus, here, we present factors associated with human–bat interactions and the first whole-genome analysis of rabies virus (RABV) circulating in human and domestic animal (dogs and cattle) hosts in Malawi.

## 2. Materials and Methods

### 2.1. Study Site and Population

**Figure 1:**
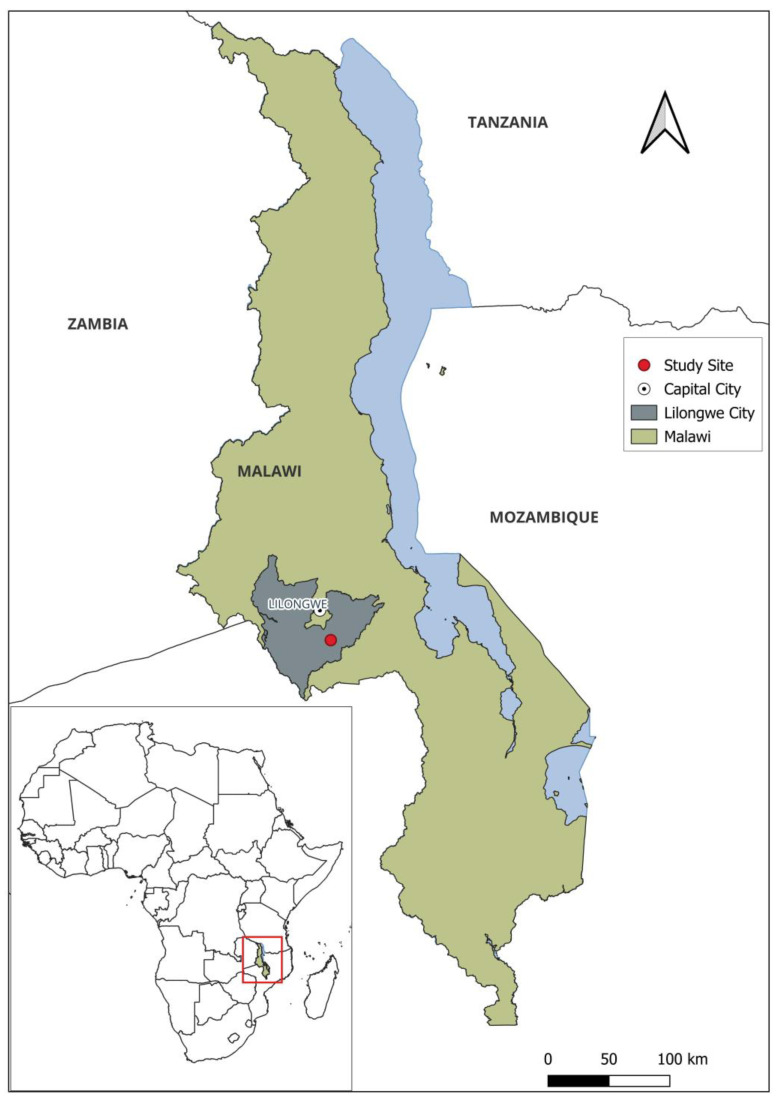
Map of Malawi showing the study site where archived samples were collected, as well as locations for bat sampling and community survey.

Situated in the southeastern region of Africa, Malawi is a landlocked country sharing borders with Mozambique, Tanzania, and Zambia. Data were collected within Lilongwe, the capital city, in February 2023. RNA samples were taken from wildlife, specifically insectivorous bats. Further, knowledge, attitude, and practices data were obtained through a semi-structured questionnaire in a rural community within Lilongwe situated at 14°08′ S, 33°51′ E (Figure 1). At the centre of this rural dwelling area is Ngala Hill, a notable landmark and tourist destination that serves as a habitat for a bat colony. The surrounding landscape consists of a small village, farmlands, and scattered homesteads, primarily inhabited by smallholder farmers who rely on crops, livestock, and some hunting for their livelihoods.

### 2.2. Study Design and Sampling

This was a cross-sectional study integrating an epidemiological survey and genomic analysis. This study assessed human–bat interactions and knowledge and practices related to rabies. In addition, the genetic diversity of RABV in humans, domestic animals, and wildlife hosts was analysed to understand vectorial capacity and transmission dynamics.

Given the relatively small size of the community (*N* = 213), the calculated sample size using Yamane’s formula [21] was *n* = 139. Participants for the community survey were recruited randomly based on being 18 years or older, residing near bat roosts, and having a willingness to participate in this study. Participants who could not provide informed consent, had cognitive impairments, or did not reside in the community were excluded.

With approval from the Department of National Parks and Wildlife in Malawi, a total of 23 insectivorous bats from the *Miniopteridae* family were sampled. This was performed in consideration of bat conservation efforts, ensuring minimal disruption to the bat population. We initially planned to sample 10% of the bat population, but the Directorate of Animal Health and Livestock Development (DALHD) in Malawi emphasised the use of 3R principles (Replacement, Reduction, and Refinement), thus limiting sampling to fewer than 30 per trip to minimize disruption. Since tissue replacement was not feasible for lyssavirus detection, we maximized Reduction by targeting bats likely diseased and unable to migrate.

### 2.3. Data Collection Procedure

Trained research assistants conducted face-to-face interviews using a structured questionnaire with 22 questions on human–bat interactions. Prior to data collection, coauthors pre-validated it for clarity, relevance, and reliability. Content validity was assessed to ensure alignment with study objectives. A pilot study was conducted with five veterinary students and five community members excluded from the final analysis.

Data collection occurred during the rainy season, when many households were female-headed and men often migrated for work. This likely contributed to the high proportion of female respondents and a lower overall response rate.

The bats were captured using mist nets and euthanised using chloroform, and their brain tissues were extracted. Three archived RABV-positive brain samples from a dog (2019), a human (2021), and a cow (2021) were retrieved from the Central Veterinary Laboratory (CVL) in Lilongwe for molecular characterisation.

### 2.4. DNA Extraction and RT-PCR Screening of Bat Samples

Total RNA was extracted from archived RABV-positive samples and individual bat brain homogenates using a QIAamp viral RNA Mini Kit (QIAGEN, Hilden, Germany) according to the manufacturer’s instructions. To detect lyssavirus in the local bat population, a one-step RT-PCR kit (Takara, Shiga, Japan) was employed to amplify the 3′ terminus of the nucleoprotein with primers RabForPyro (5-AACACYYCTACAATGGA-3) and RabRevPyro-biot 1–3 (5-CCAATTNGCACACATTT TGTG-3) [22]. Five microlitres of eluted RNA was added to a 45 µL master mix with a final volume of 50 µL. The PCR conditions included a reverse transcription step at 50 °C for 30 min, followed by an initial denaturation at 94 °C for 2 min. This was followed by 45 cycles at 94 °C for 30 s, annealing at 52 °C for 30 s, and extension at 72 °C for 30 s, with a final extension at 72 °C for 5 min. The resulting amplicons were visualized using a 1.5% agarose gel stained with ethidium bromide with a 100 bp DNA ladder as a size marker.

### 2.5. Whole-Genome Sequencing of Rabies Virus

For whole-genome sequencing of positive rabies RNA, samples were reverse-transcribed into cDNA with M-MLV RTase (Promega, Madison, WI, USA), after which cDNA was subjected to tiling PCR and PCR cleanup as described by Brunker et al., 2020 [23]. Purified amplicons were then subjected to library preparation using a Nextera XT DNA sample preparation kit (Illumina, San Diego, CA, USA) as per the manufacturer’s instructions. The prepared libraries were then sequenced on an Illumina MiSeq with 75 bp pair-end reads. Sequence reads from each sample were assembled into a single contig using Qiagen CLC Genomics Workbench 21.0 (QIAGEN, Aarhus, Denmark) to produce whole-genome consensus sequences for downstream analysis.

## 3. Data Analysis

### 3.1. Epidemiological Data Analysis

The data from the questionnaire underwent univariate analysis for descriptive statistics and bivariate analysis, assessing the association with bat contact. Additionally, an affirmative response (“Yes”) was considered as knowledge or positive practice. The total number of affirmative responses were tallied together and expressed as percentages. Variables with *p* ≤ 0.250 in bivariate analysis were included in the multivariate logistic regression model, where backward stepwise elimination was applied to identify significant predictors, at *p* < 0.05. The primary outcome variable was bat contact (Yes/No). Model fit was confirmed by Hosmer–Lemeshow (*p* > 0.5) and Omnibus tests (*p* < 0.01).

### 3.2. Sequence Alignment and Phylogenetic Analysis

RABV sequences from various global regions, lineages, and phylogroups were retrieved from GenBank and aligned with the study sequences using Clustal Omega v1.2.4 [24] to identify lineages and diversity of RABV in Malawi. Introns and other non-coding regions within the multiple sequence alignment were removed, and the remaining complete genes were concatenated into sequences of 10,860 bp using a custom Python script (Python v3.11). The resulting alignment was then converted to amino acid sequences and viewed in NCBI MSA Viewer “https://www.ncbi.nlm.nih.gov/projects/msaviewer/ (accessed on 25 November 2024)”. In addition, phylogenetic analysis was performed using IQ-TREE with the General Time Reversible (GTR) model, incorporating invariant sites and a gamma distribution as determined by the model test incorporated in IQtree [25]. Branch support was assessed with SH-like aLRT (1000 replicates).

## 4. Results

### 4.1. Sociodemographic Characteristics of Study Participants

This study enrolled 72 participants, with 81% (58/72) being female. Educational background varied; 10% (7/72) had no formal education, 60% (43/72) completed primary, 23% (16/72) finished secondary, and 7% (5/72) attained tertiary education (Table 1).

### 4.2. Reported Human–Bat Interactions

Half of the participants, 50% (36/72), ate bats, and 6% used bat caves for traditional medicine. Additionally, 46% (32/72) reported bats entering their living dwellings, and 21% (15/72) observed bats in their ceilings. Furthermore, 43% (31/72) of respondents reported using no protective equipment when handling dead or live bats (Table 2).

### 4.3. Knowledge Related to Human–Bat Interactions

Only 28% (20/72) of the participants knew bats posed a risk of infectious diseases, 24% (17/72) knew bats can get sick, and 50% (36/72) recognised the danger of encountering sick bats (Table 3). The average knowledge score was 67 ± 15 standard deviation, with 57% of the participants scoring below the mean (Table 4).

### 4.4. Practices Related to Human–Bat Interactions

Fifty-eight percent (36/72) of the participants reported washing their hands with soap or disinfectant after a bat scratch. Eighty-two percent (51/72) of the participants reported seeking medical attention after a bat scratch or bite. Additionally, 39% (23/72) of the participants reported vaccinating their dogs against rabies. Furthermore, 55% practised improper disposal, such as open dumping, feeding bats to dogs, or consumption, while 51% did not disinfect the environment with disinfectant (Table 5).

The overall practice score was 73 ± 19 standard deviation, and 54% of participants showed good practices. Within the good practice category, 57% were 36 years and above, and 80% were married. A statistical difference was observed in daily income (*p* = 0.022); among those with poor practice, 52% earned less than MWK 2500 (USD 1.50) compared to 80% in the good practice group (Table 6).

### 4.5. Analysis of Risk Factors Associated with Human–Bat Interactions

A notable correlation existed between bats entering dwellings and bat contact (χ^2^ = 10.672, *p* = 0.02). Furthermore, a significant association was observed between the duration of residence and bat consumption (*χ*^2^ = 7.342, *p* = 0.028) and between occupation and bat consumption (χ^2^ = 7.432, *p* = 0.014) (Table 7). Individuals with a residence duration of fewer than 5 years exhibited a significantly higher likelihood of bat consumption compared to those with a longer duration of residence (OR: 8.00, CI 1.05–60.72, *p* = 0.028). Conversely, the odds of consuming bat meat were markedly higher among participants in occupations other than farming (OR: 9.0, CI 1.2–67.4, *p* = 0.014).

In the stepwise logistic regression with three independent variables, age, the locations of bat roosts, and bats entering dwellings accurately fit the generated model. The Hosmer–Lemeshow test revealed no significant lack of fit in the model (*p* > 0.8), indicating a good fit. The Omnibus Test of Model Coefficients indicated that the overall model was statistically significant (*p* < 0.01), suggesting that the included independent variables had a significant impact on predicting the outcome variable. Bats entering dwellings were the sole statistically significant predictor of the outcome of bat contact. Participants who responded “No” to the question about bats entering dwellings (aOR: 0.075, CI 0.008–0.682, *p* = 0.021) were significantly less likely to encounter bats than the reference category (Table 8).

### 4.6. Amplification of Lyssavirus RNA in Wildlife Hosts

All twenty-three brain samples from *Miniopteris natalensis* bats tested negative for lyssavirus RNA using RT-PCR.

### 4.7. Phylogenetic Analysis of Whole-Genome Sequences of Lyssavirus

**Figure 2:**
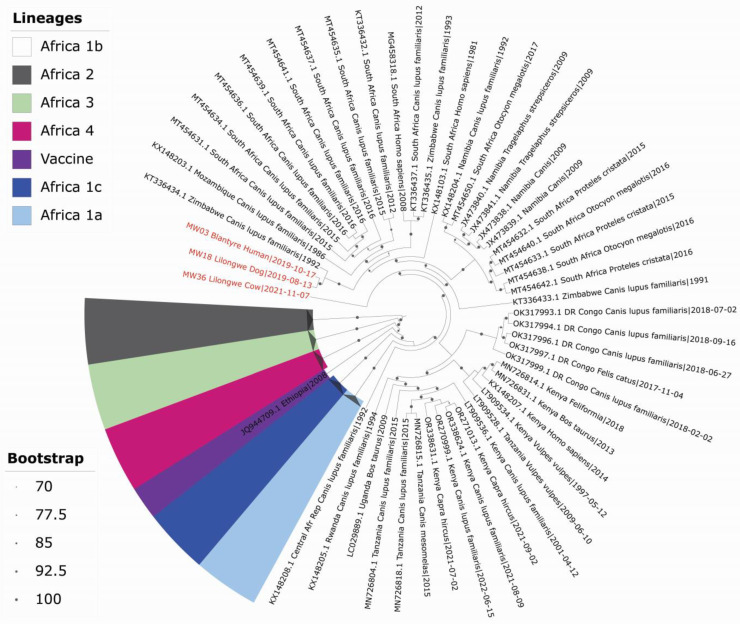
Maximum likelihood phylogenetic tree of Malawian RABV and reference strains. The tree, generated using IQ-TREE with the GTR+F+I+G4 model and 1000 bootstraps, is based on 10,860 bp coding sequences. All RABV strains are colour-coded, with Malawian sequences from this study labelled in red.

Whole-genome phylogeny using nucleotide sequences revealed that the sequences of RABVs from Malawi belong to the Africa 1b subclade, Phylogroup I, with high-level sequence similarity (≥95%), regardless of host species and geographic origin. The study sequences clustered closely with Zimbabwean, Mozambiquan, Namibian, South African, and Tanzanian Dog sequences, with strong support from significant bootstrap values (>97%). MW36_Lilongwe_Cow/2021 appeared higher in the tree, while MW18_Lilongwe_Dog/2019 and MW03_Blantyre_Human/2019 clustered together, indicating shared ancestry (Figure 2).

### 4.8. Characterisation of RABV from a Human, Cow, and Domestic Dog

The three whole-genome sequences, MW18_Lilongwe_Dog/2019, MW03_Blantyre_Human/2019, and MW36_Lilongwe_Cow/2021, were 11,801 nucleotides (nt) long. The mRNA and coding sequences of each gene were analysed, characterised, and organised as follows; N (1353 nt), P (894 nt), M (639 nt), G (1575 nt), and L (6384 nt). At the whole-genome level, sequence MW18_Lilongwe_Dog/2019 shared (95–97%) nucleotide identity with complete genomes from South Africa, Zimbabwe, and Tanzania (Supplementary Table S1). Similarly, MW03_Blantyre_Human/2019 and MW36_Lilongwe_Cow/2021 showed (95–98%) and (96–97%) nucleotide identity, respectively. At the amino acid level, MW03_Blantyre_Human/2019 and MW36_Lilongwe_Cow/2021 showed 99% and 92% similarity with MW18_Lilongwe_Dog/2019, respectively.

#### Features of the Structural Proteins of RABV from Human, Cow, and Domestic Dog

The N gene of the study sequences encoded a protein of 450 amino acids. Two unique substitutions (Ala_123_ and Lys_254_) were found in all three sequences, and two (Iso_322_ and Asp_448_) were unique to MW36_Lilongwe_Cow/2021. The RNA-binding domain at sites 298–352 was conserved apart from a single amino acid substitution Iso_322_ to Val_322_ in MW36_Lilongwe_Cow/2021. A casein-type phosphorylation site [26], Ser_389_, linked to regulating viral RNA transcription and replication [27], was conserved. Additionally, antigenic site I (358–367), a B-cell epitope [28], and antigenic site IV (359–366 and 375–383) were also conserved.

Four unique substitutions (Glu_71_, Thr_72_, Met_104_, and Pro_140_) in the 297-amino acid P gene were identified in MW18_Lilongwe_Dog/2019 and MW03_Blantyre_Human/2019, with Glu_71_ and Thre_72_ located in the Variable Domain 1 (VD1) region and Pro_140_ within the VD2. The first 19 amino acids (aa) of the Conserved Domain 1 (CD1), which have transcriptional roles when interacting with L, were conserved [14], along with the entire CD1 and CD2 domains. The motifs encoded as KSTQT (aa 144–148) and FSKKYKF (aa 209–215), responsible for interacting with and binding to the dynein light chain, were also conserved [4,29].

With regard to the M gene consisting of 212 aa, study sequences had 10 more amino acids at the N-terminus than other sequences from India, Nishigara RC EH, and DRV China strains. The M gene from study sequences shared 85% amino acid identity with Australian bat lyssavirus (ABLVB) [30], which also has a 212 aa M gene. Compared to ABLVB, two unique (Lys_2_ and Gly_5_) substitutions were identified in the first 10 aa. When all the strains were compared, two unique substitutions (Leu_178_ and Asp_202_) were found across the entire M gene sequence. The proline-rich motif PPEY (aa 45–48) and the essential site Glu_68_, a key regulator for RNA synthesis [31], were both conserved.

**Figure 3:**
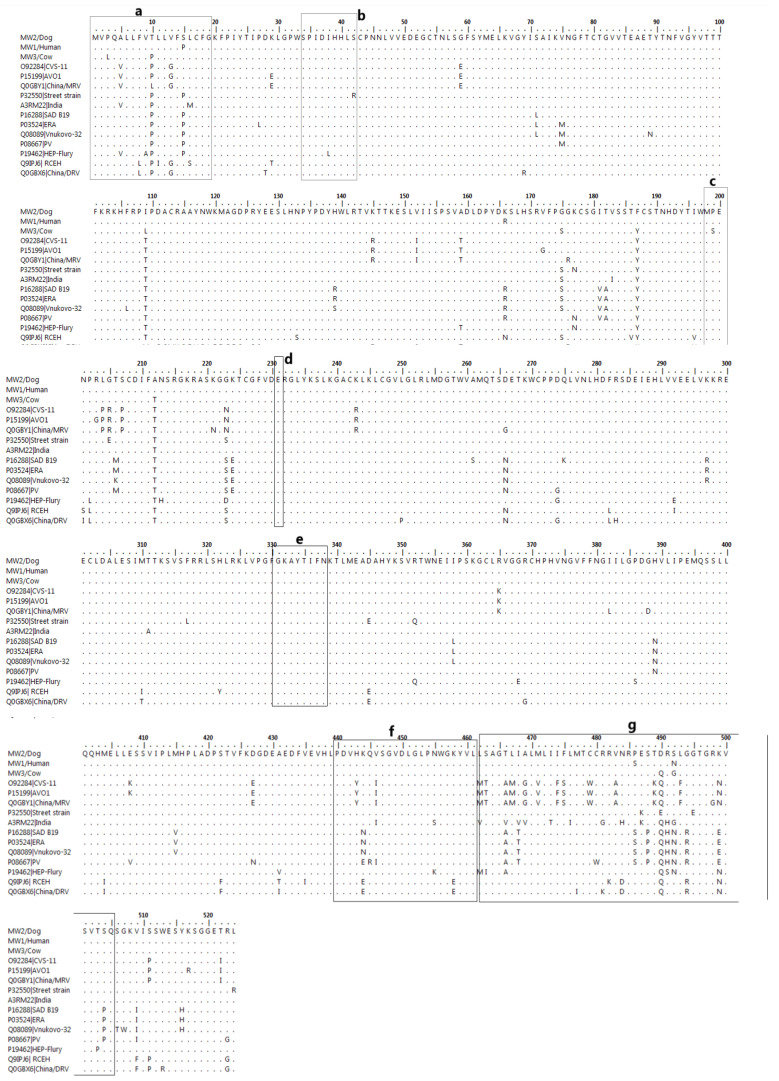
Multiple sequence alignment of the G amino acid sequence. (**a**): Signal peptide, (**b**,**c**): antigenic site II, (**d**,**e**): antigenic site I and III, (**f**,**g**): transmembrane and cytoplasmic domain.

The G gene (524 aa), responsible for viral attachment, showed conserved antigenic sites (I, II, III, IV, and a) except for IIb in MW36_Lilongwe_Cow/2021, where a P199S substitution was observed. Several unique substitutions (Thr_10_, Ile_109_, Ala_212_, Gly_223_) were identified, including Arg_166_ in MW18_Lilongwe_Dog/2019. Within the cytoplasmic domain, Glu_490_ and Glu_499_ were substituted by Asp_490_ and Lys_499_, respectively (Figure 3).

The L gene of the three study sequences encoded a protein of 2127 amino acids. Unique substitutions were identified at the following sites: Ile_449_, Asn_878,883_, Thr_1136_, Val_1172_, Val_1806_, and Cys_1825_. Glu_1138_, Ser_1140_, and Ser_1318_ were only present in MW18_Lilongwe_Dog/2019 and MW36_Lilongwe_Cow/2021. A P1093A substitution was observed in all MW18_Lilongwe_Dog/2019 and MW03_Blantyre_Human/2019, while an L1093A was in MW36_Lilongwe_Cow/2021. Additionally, a Thre_1204_ was substituted with Ile_1204_. The following regions were conserved: the GHP motif (aa 372–374), Domain II (aa 544–563) for template recognition [21], Domain III (728–731) for RNA polymerase activity, and Domain VI (aa 1704–1709), a glycine-rich motif (GXGXXG) involved in protein kinase activity [3].

## 5. Discussion

Rabies remains a major public health challenge, with dogs as the primary source of infection. However, bats are increasingly recognised as reservoirs for zoonotic pathogens, including lyssaviruses, with human–bat interactions driving pathogen spillover events [32,33]. This study investigated human–bat interactions, including related knowledge and practices, in a rural Malawian community. We also investigated lyssavirus in bats and characterised rabies virus from humans and domestic hosts using WGS. By combining community-level insights and genomic analysis, this study sought to enhance the understanding of rabies epidemiology and inform effective prevention and control strategies. The findings further aim to support global rabies control efforts and advance One Health approaches to combat zoonotic diseases.

The present study revealed that half of the respondents reported various forms of interaction with bats, including using bat caves for traditional medicine and consuming bats. However, less than half were aware that bats can be a source of zoonotic infections. These findings align with a study conducted in Tanzania, where 57.5% (187/325) of participants reported contact with bats, yet only 4% believed bats could transmit rabies [34]. Similar observations were made in Kenya and Guatemala, with the latter reporting that 90% of respondents had little to no knowledge about rabies in bats despite the documented presence of rabies in the country [35,36]. Bat-mediated rabies exposure, although rare, has resulted in fatalities in Scotland [37], Kenya [35], and South Africa [38].

Multiple studies conducted across Africa have consistently documented a pattern of human-driven interactions with bats primarily through hunting for consumption and traditional medicine [39,40,41,42].The human–bat interactions observed in this study further align with the literature, particularly a study by Wright et al., where it was reported that global patterns of exposure to bats are intentional and often associated with household activities [43]. Education emerged as an important factor in shaping knowledge and behaviours, as evidenced by participants demonstrating greater awareness and adopting safer practices, consistent with observations from studies conducted in Malawi [44], Bangladesh [45], and South Korea [46]. Furthermore, significant gaps in knowledge and practices related to human–bat interactions, with occupation identified as a key contributing factor, have been corroborated by findings from numerous studies conducted across Africa and Asia [16,47,48].

The current study shows that respondents who reported bats not entering their dwellings were less likely to have interactions with bats. This implies that the presence of bats inside dwellings significantly increases the likelihood of bat contact, while the absence of bats indoors is a protective factor against bat contact. This finding agrees with a study conducted in a rural part of Kenya where the use of the same building by humans and bats led to frequent interactions, hence increasing the risk of pathogen spillover [49]. Another study in Sierra Leone indicated that human activities, such as residing in proximity to bat roosts or within structures housing bats, elevated the likelihood of human–bat interactions, thus raising the possibility of exposure to bat pathogens [50]. These findings highlight the importance of implementing measures of sealing any bat entry points in homes to reduce these interactions and mitigate health risks. Adopting a One Health approach, which integrates efforts across human, animal, and environmental health sectors, could enhance surveillance, promote community education, and improve collaboration to address the observed risk factors and prevent potential spillover events.

A study conducted in North Africa did not detect lyssavirus in the bat samples [11]. Similarly, in another study in Belgium, no lyssavirus was detected in bat saliva samples [51]. These findings corroborate the results from the present study in which no lyssavirus genome was detected in bat samples by RT-PCR. On the contrary, a study in Spain demonstrated lyssavirus antibodies in blood pellets, and positive results were further demonstrated using RT-PCR on brain and blood samples [52]. The lack of detection of lyssavirus in bat samples could be attributed to this study’s limited small sample size. Detecting lyssaviruses can be challenging, as infections might be sporadic, and viral loads may fluctuate, leading to potential underestimation of prevalence in bat populations [51]. The sporadic and fluctuating nature of lyssavirus in bats suggests variable rabies transmission risk. Studies assessing bat-to-human transmission rates are recommended and essential for accurately quantifying this risk. In addition, sampling took place during the rainy season, marked by cool to moderate temperatures and *Miniopteris natalensis* bats approaching winter hibernacula. Previous studies have noted the seasonal influence of rabies occurrence in bats, with higher rates reported during the dry season [39,53]. The immigration of rabid bats into a colony during the dry season and the fluctuating and cyclical patterns of rabies infection in bat populations have been observed in Cameroon and Brazil [54,55]. These observations suggest that rabies detection studies may be more effective during the dry season when viral activity and transmission risk are likely higher.

The nucleoprotein is highly conserved in any RABV strain. Critical sites, including Ser_389,_ a casein-type phosphorylation and antigenic site I, a B-cell epitope, and IV, were all conserved in the Malawian strains and other African RABV strains [56], suggesting evolutionary constraints shaped by host–virus interactions common in this geographic region. In addition, the RNA-binding domain (amino acids 298–352) of the MW36_Lilongwe_Cow/2021 strain exhibited an isoleucine-to-valine substitution at position 322 (Iso322Val), a mutation also observed in European bat lyssavirus 2 (EBLV-2; GenBank #A4UHQ3), PV (GenBank #P06025), and the Chinese MRV strain (GenBank #Q0GBY4), suggesting convergent evolution driven by environmental or host pressures. This substitution is noteworthy for its potential implications in adaptive evolution, as it may enhance RNA binding or replication efficiency under specific ecological conditions and in cross-species infection, as similar changes across lyssaviruses point to adaptations that could influence host range and zoonotic potential [57,58].

The presence of Glu71 and Thr72 in Variable Domain 1 (VD1) and Pro140 in Variable Domain 2 (VD2) highlights distinct evolutionary trajectories in the Malawian strains, with potential implications for functional specialisation, such as roles in immune evasion or host factor interactions and for pathogenicity and host range, as changes in the phosphoprotein (P gene) may influence interactions with host cellular machinery, particularly interferon antagonism. The Malawian RABV strains encoded a 212 aa M gene, 10 amino acids longer than the Indian, RC EH, and DRV China strains. The Malawian strains also shared 85% similarity with an Australian bat lyssavirus (Q9QSP2) isolated from an insectivorous bat [30]. This similarity might suggest that both viruses have maintained these extra amino acids to adapt to similar selection pressures despite geographical and host differences [57,58]. Further comparative studies could clarify the functional role of these additional amino acids in the matrix protein.

The glycoprotein is essential for facilitating virus–host interactions, influencing both pathogenicity and the ability to cross species barriers [6]. It also triggers the immune system to produce neutralising antibodies, vital for protective immunity against the virus [59]. Given this importance, the glycoprotein has been a focal point in molecular epidemiological studies [60]. The Malawian strains showed several unique substitutions along the G gene including antigenic site IIb and the cytoplasmic domain. These substitutions may alter how effectively vaccines can induce an immune response, highlighting the need for further research to continuously monitor these substitutions and understand their impact on vaccine efficacy [59] as well as their ability to enhance the strains to cross species barriers, thus posing additional challenges for public health.

The L protein, essential for transcription and replication [2], exhibited conserved motifs in the Malawian strains, including the GHP motif (amino acids 372–374), Domain II (amino acids 544–563) for template recognition, Domain III (amino acids 728–731) for RNA polymerase activity, and Domain VI (amino acids 1704–1709), a glycine-rich motif (GXGXXG) associated with protein kinase activity [3]. The conservation of these regions underscores their critical roles in viral replication and polymerase function, highlighting their potential as targets for antiviral development to inhibit RABV replication across strains and provide phylogenetic insights into the genetic relatedness and evolutionary constraints among RABV strains.

A phylogenetic analysis using complete genome sequences from multiple countries and Malawi corroborated the results of a recent molecular epidemiological study on RABV in Malawi using partial N gene sequences [14] and aligns with the observation that viruses from the same geographical area cluster together [56,61]. In addition, MW03_Blantyre_Human/2019 and MW36_Lilongwe_Cow/2021 shared 99% and 92% amino acid similarity with MW18_Lilongwe_Dog/2019, consistent with a previous study from Zambia and Zimbabwe where samples clustered closely despite differing hosts and regions [57]. Similarly, a study in Jordan identified a viral dog genome with >98.8% similarity to a virus from Israeli cattle [62]. This highlights the critical role of domestic dogs in the RABV transmission cycle. In the context of Malawi and its neighbours, the similarity of cultures as well as the absence of physical barriers provides ease of movement of humans and animals across borders, potentially contributing to cross-border transmission of pathogens [14]. Owing to the low sample size of sequences used in this study, this aspect could not be assessed but sets the foundation for future studies. This emphasises the importance of the One Health approach and regional cooperation in rabies control and further suggests a need for coordinated multisectoral surveillance and vaccination programs to effectively prevent rabies spread in the region.

This study had a few limitations: the community survey had a low response rate and predominantly female participation. The data collection took place during the rainy season, and most of the community’s residents are farmers who engage in temporary labour; most households were female-headed, as the men were away. These may affect generalisability and representativeness; however, findings remain relevant for similar settings and should be considered with care when extrapolating to broader regions.

## 6. Conclusions

This study, despite failing to detect lyssavirus in bat samples collected from rural Lilongwe, offers critical insights into human–bat interactions, zoonotic risks, and the molecular characterisation of RABV in Malawi. It identifies significant gaps in public awareness and risky behaviours that facilitate zoonotic disease transmission, highlighting the need for targeted education and community-level interventions. The genetic analysis of Malawian RABV strains underscores the complexity of viral evolution and control, with conserved functional domains playing a role in viral fitness and unique substitutions potentially influencing host interactions and immune responses. These findings stress the importance of regional surveillance to monitor RABV and related lyssaviruses, the development of region-specific vaccines to address strain-specific variations, and the adoption of a One Health approach to mitigate cross-species and regional transmission risks. Further research into genetic traits and transmission dynamics remains vital for improving rabies control strategies and strengthening public health initiatives in Malawi and beyond.

## Figures and Tables

**Table 1:** Summary of sociodemographic information.

Variable	Category	Number	Proportion (%)
Sex	Female Male	58 14	81 19
Age	<36 ≥36	39 32	54 46
Education	None Primary Secondary Tertiary	7 43 16 5	10 60 23 7
Marital status	Married Single/divorced	18 54	25 75
Family size	<5 members ≥5 members	57 15	79 21
Occupation	Farmers Govt/private	62 10	86 14
Daily income	<MWK 2500 (USD 1.50) ≥MWK 2500 (USD 1.50)	50 8	86 14
Length of residence	<5 years ≥5 years	9 63	13 87

**Table 2:** Summary of human–bat interactions among respondents.

Variable	Category	Number	Proportion (%)
Touched bats	Yes No	11 56	16 84
Bat consumption	Yes No	36 36	50 50
Bat caves for traditional medicine	Yes No	4 67	6 94
Bats coming inside dwellings	Yes No	32 38	46 54
Bats are seen in the ceiling	Yes No	15 57	21 79
Bats are seen in trees near my dwelling	Yes No	19 53	26 74
Equipment for handling bats	None Gloves	31 23	57 43

**Table 3:** Knowledge associated with human–bat interactions.

Knowledge Variables	Category	Number	Proportion (%)
Bats as a source of infectious diseases	Yes No	20 51	**28** 72
Knew that sick/dead bats are dangerous to come in contact with	Yes No Don’t know	36 16 19	**51** 22 27
Knew that bats get sick	Yes No Don’t know	17 13 40	**24** 19 57
Heard about bat conservation	Yes No	3 66	**4** 96
Knew that some bats eat fruits	Yes No	31 41	**43** 57
Knowledge on vaccines	Yes No	25 46	**35** 65
Knew that educating people can prevent bat-related diseases	Yes No	59 11	**84** 16

Correct responses are indicated in bold.

**Table 4:** Knowledge distribution across demographic factors.

Knowledge					
Demographic Variables		Low	High	*χ* ^2^	*p* Value
		N (%)	N (%)		
Overall		41 (57)	31 (43)		
Gender	Female Male	36 (88) 5 (12)	22 (71) 9 (29)	3.195	0.074
Age	<36 years ≥36 years	20 (50) 20 (50)	17 (59) 12 (41)	0.502	0.478
Educational level	<Secondary ≥Secondary	30 (73) 11 (27)	20 (67) 10 (33)	0.352	0.553
Marital status	Single	12 (29)	6 (19)	0.925	0.336
	Married	29 (71)	25 (81)		
Duration of residence in area	<5 years	5 (12)	4 (13)	n/a	0.928
	≥5 years	36 (88)	27 (87)		
Occupation	Farmers	36 (88)	26 (84)	0.228	0.633
	Others	5 (12)	5 (16)		
Daily income	<MWK 2500 (USD 1.50)	30 (66)	20 (74)	0.970	0.325
	≥MWK 2500 (USD 1.50)	15 (33)	7 (26)		

n/a = the minimum expected count was <5, a Fischer’s exact test was used instead, χ^2^ = chi-square.

**Table 5:** Practices related to human–bat interactions.

Practice Variables	Category	Number	Proportion (%)
Wash fruits before eating	Yes No	66 6	**92** 8
Wash using soap after bat scratch or bite	Yes No	36 26	**58** 42
If you get scratched, where would you go?	Hospital Nowhere	51 11	**82** 18
Wash hands using soap after handling bats	Yes No	41 26	**61** 39
Method of disposing of a dead bat	Proper Improper	28 23	**55** 45
Disinfecting environment after bat disposal	Yes No	33 35	**49** 51
Dog vaccination against rabies	Yes No	23 36	**39** 61

Good practice indicated in bold.

**Table 6:** Practice distribution across sociodemographic factors.

Practice					
Demographic Variables		Poor	Good	*χ* ^2^	*p* Value
		N (%)	N (%)		
**Overall**		33 (46)	39 (54)		
Gender	Female	27 (82)	31 (80)	0.062	0.803
	Male	6 (18)	8 (20)		
Age	<36 years	21 (66)	16 (43)	3.457	0.063
	≥36 years	11 (34)	43 (57)		
Educational level	<Secondary	21 (64)	29 (76)	1.363	0.243
	≥Secondary	12 (36)	9 (24)		
Marital status	Single	10 (30)	8 (20)	0.914	0.339
	Married	23 (70)	31 (80)		
Duration of residence in area	<5 years	5 (15)	4 (10)	n/a	0.531
	≥5 years	28 (85)	35 (90)		
Occupation	Farmers	28 (85)	34 (87)	n/a	0.776
	Others	5 (15)	5 (13)		
Daily income	<MWK 2500 (USD 1.50)	22 (52)	28 (80)	5.240	0.022
	≥MWK 2500 (USD 1.50)	20 (48)	7 (20)		

n/a = the minimum expected count was <5, a Fischer’s exact test was used instead, χ^2^ = chi -square.

**Table 7:** Bivariate analysis of bat consumption.

Variable	Number	Test Static	*p*-Value
Education			
<Secondary level	50	1.497 ^a^	0.3
≥Secondary level	21		
Age			
<36 years old	37	4.176 ^a^	0.55
≥36 years old	32		
Duration of residence			*
<5 years	9	6.222 ^b^	**0.028**
≥5 years	63		
Occupation			*
Farmer	62	7.432 ^a^	**0.014**
Other	10		
Daily income			
<MWK 2500	50	1.424 ^b^	0.272
≥MWK 2500	8		
Location of bat roosts			
Ceiling/trees nearby	31	2.837 ^a^	**0.148**
Other places	39		
Bats coming inside houses			
Yes	38	0.230 ^a^	0.811
No	32		
Do you dislike bats?			*
Yes	42	4.799 ^a^	**0.041**
No	24		

^a^ = Chi-square value, ^b^ = Fischer exact value, boldface = considered for multivariate analysis (cut-off *p* < 0.250), * = statistical significance at *p* < 0.05.

**Table 8:** Risk factors associated with bat contact (logistic regression).

	Unadjusted			Adjusted		
	Odds Ratio	95% CI	*p*-Value	Odds Ratio	95% CI	*p*-Value
Age						
<36 years	Ref			Ref		
≥36 years	0.40	0.047–1.239	0.092	0.19	0.127–1.979	0.075
Location of bat roosts						
Ceiling	Ref					
Other places	0.35	0.092–1.353	0.129	0.35	0.08–1.53	0.067
Bats coming inside dwellings						
No	Ref					
Yes	0.40	0.275–0.605	0.002 *	0.075	0.008–0.68	0.021 *

* Statistically significant at a 5% significance level, CI = Confidence Interval, Ref = reference category.

## Data Availability

The datasets used and/or analysed during the current study are available from the corresponding author upon reasonable request.

## Supplementary Materials

The following supporting information can be downloaded at: https://www.mdpi.com/article/10.3390/tropicalmed10040095/s1, Supplementary Table S1. BLAST results for Malawian lyssavirus sequences.

## Abbreviations

The following abbreviations are used in this manuscript:

MDPI	Multidisciplinary Digital Publishing Institute
DOAJ	Directory of open access journals
TLA	Three letter acronym
LD	Linear dichroism
RRVs	Rabies-related lyssaviruses
RT-PCR	Reverse Transcriptase Polymerase Chain Reaction
PCR	Polymerase Chain Reaction
RABV	Rabies virus
OR	Odds Ratio
aOR	Adjusted Odds Ratio
RNA	Ribonucleic acid
cDNA	Complimentary deoxyribonucleic acid
DNA	Deoxyribonucleic acid
WGS	Whole-genome sequencing
MW	Malawi
MSA	Multiple sequence alignment
Aa	Amino acid
