# Correction: Singano et al. One Health Lens on Rabies: Human–Bat Interactions and Genomic Insights of Rabies Virus in Rural Lilongwe, Malawi. *Trop. Med. Infect. Dis.* 2025, *10*, 95

**DOI:** 10.3390/tropicalmed10050133

**Published:** 2025-05-15

**Authors:** Nathan Singano, Henson Kainga, Elisha Chatanga, Joseph Nkhoma, Gilson Njunga, Julius Chulu, Rabecca Tembo, Hirofumi Sawa, Walter Muleya

**Affiliations:** 1 Department of Biomedical Sciences, School of Veterinary Medicine, The University of Zambia, Lusaka P.O. Box 32379, Zambia; singanonathan@gmail.com; 2 Department of Veterinary Epidemiology and Public Health, Faculty of Veterinary Medicine, Lilongwe University of Agriculture and Natural Resources, Lilongwe P.O. Box 219, Malawi; hensonkainga@luanar.ac.mw; 3 Department of Veterinary Pathobiology, Faculty of Veterinary Medicine, Lilongwe University of Agriculture and Natural Resources, Lilongwe P.O. Box 219, Malawi; echatanga@luanar.ac.mw; 4 Central Veterinary Laboratory (CVL), Lilongwe P.O. Box 527, Malawi; joemnkhoma@gmail.com; 5 Department of Animal Health and Livestock Development, Lilongwe P.O. Box 2096, Malawi; gnjunga@tappmalawi.org (G.N.); juliuschulu09@gmail.com (J.C.); 6 Trustees of Agricultural Promotion Programme, P/Bag A21, Lilongwe P.O. Box 2096, Malawi; 7 Department of Pathology and Microbiology, School of Medicine, The University of Zambia, Lusaka P.O. Box 50110, Zambia; rabecca.tembo@unza.zm; 8 International Institute for Zoonosis Control, Hokkaido University, Sapporo 001-0020, Japan; h-sawa@ivred.hokudai.ac.jp; 9 Institute for Vaccine Research and Development, Hokkaido University, Sapporo 001-0021, Japan

There was an error in the original publication [1]. A previous proofreading version of the manuscript, which had not incorporated minor text revisions and additional clarifications, was mistakenly submitted.

A correction has been made to multiple sections, Introduction, Materials and Methods, and Results, with minor text updates across several paragraphs to improve clarity and accuracy.

Abstract: RABV genomes from human.

Section 1 (Paragraph 2): RABV in Africa is categorised into lineages 1a, 1b, 2, 3, and 4.

Section 1 (Paragraph 3): children are the most affected.

Section 1 (Paragraph 4): Thus, here, we present factors associated with human–bat interactions and the first whole-genome analysis of rabies virus (RABV) circulating in human and domestic animal (dogs and cattle) hosts in Malawi.

Section 2.4: The PCR conditions included a reverse transcription step at 50 °C for 30 min, followed by an initial denaturation at 94 °C for 2 min. This was followed by 45 cycles at 94 °C for 30 s, annealing at 52 °C for 30 s, and extension at 72 °C for 30 s, with a final extension at 72 °C for 5 min. The resulting amplicons were visualized using a 1.5% agarose gel stained with ethidium bromide with a 100 bp DNA ladder as a size marker.

Section 4.7: Africa 1b subclade.

Section 4.7: The study sequences clustered closely with Zimbabwean, Mozambiquan, Namibian, South African, and Tanzanian Dog sequences, with strong support from significant bootstrap values (>97%). MW36_Lilongwe_Cow/2021 appeared higher in the tree, while MW18_Lilongwe_Dog/2019 and MW03_Blantyre_Human/2019 clustered together, indicating shared ancestry (Figure 2).

We have also added district names to the Malawian sequences throughout the manuscript i.e., from MW03_Human/2019 to MW03_Blantyre_Human/2019.

Section 5 (Paragraph 7 and Paragraph 8): changed MW strains to Malawian strains.

The authors state that the scientific conclusions are unaffected. This correction was approved by the Academic Editor. The original publication has also been updated.
