# Supplementary material for: One Health Lens on Rabies: Human–Bat Interactions and Genomic Insights of Rabies Virus in Rural Lilongwe, Malawi
Source: Trop Med Infect Dis. 2025 Apr 4;10(4):95. doi: 10.3390/tropicalmed10040095 (PMC12031134; doi:10.3390/tropicalmed10040095)
Supplement: Supplementary file 1 [file tropicalmed-10-00095-s001.zip › tropicalmed-3486336-supplementary.pdf]

data

MDPI

Data Descriptor

One Health Lens on Rabies: Human–Bat Interactions and Genomic Insights of Rabies Virus in Rural Lilongwe, Malawi

Nathan Singano [1], Henson Kainga [2], Elisha Chatanga [3], Joseph Nkhoma [4], Gilson Njunga [5,6], Julius Chulu [5], Rabecca Tembo [7], Hirofumi Sawa [8,9] and Walter Muleya [1,*]

1 Department of Biomedical Sciences, School of Veterinary Medicine, The University of Zambia, Lusaka P.O. Box 32379, Zambia; singanonathan@gmail.com
2 Department of Veterinary Epidemiology and Public Health, Faculty of Veterinary Medicine, Lilongwe University of Agriculture and Natural Resources, Lilongwe P.O. Box 219, Malawi; hensonkainga@luanar.ac.mw
3 Department of Veterinary Pathobiology, Faculty of Veterinary Medicine, Lilongwe University of Agriculture and Natural Resources, Lilongwe P.O. Box 219, Malawi; echatanga@luanar.ac.mw
4 Central Veterinary Laboratory (CVL), Lilongwe P.O. Box 527, Malawi; joemnkhoma@gmail.com
5 Department of Animal Health and Livestock Development, Lilongwe P.O. Box 2096, Malawi; gnjunga@tappmalawi.org (G.N.); juliuschulu09@gmail.com (J.C.)
6 Trustees of Agricultural Promotion Programme, P/Bag A21, Lilongwe, Malawi
7 Department of Pathology and Microbiology, School of Medicine, The University of Zambia, Lusaka P.O. Box 50110, Zambia; rabecca.tembo@unza.zm
8 International Institute for Zoonosis Control, Hokkaido University, Sapporo 001-0020, Japan; h-sawa@ivred.hokudai.ac.jp
9 Institute for Vaccine Research and Development, Hokkaido University, Sapporo 001-0021, Japan
* Correspondence: muleyawalter@gmail.com or walter.muleya@unza.zm

Abstract: Rabies, a fatal zoonotic disease, affects humans, domestic animals, and wildlife predominantly in Africa, Asia, and Latin America. In Malawi, rabies virus (RABV) is primarily transmitted by infected dogs, impacting humans and cattle. Lyssavirus has also been documented in insectivorous bats. A community survey near bat roosts assessed knowledge, attitudes, and practices regarding bat-borne zoonoses. Bat samples were tested for lyssavirus using RT-PCR, and RABV genomes from humans and domestic animals were sequenced and analysed phylogenetically. The survey revealed that 50% of participants consumed bat meat, and 47% reported bats entering their homes. Reduced bat presence indoors significantly lowered contact risk (aOR: 0.075, $p = 0.021$). All 23 bat samples tested negative for lyssavirus. Malawian RABV genomes, 11,801 nucleotides long, belonged to the Africa 1b lineage, showing >95% similarity with GenBank sequences. Phylogenetic analysis indicated close clustering with strains from Tanzania, Zimbabwe, and South Africa. Human and cattle strains shared 99% and 92% amino acid similarity with dog strains, respectively, with conserved critical sites and unique substitutions across all five RABV genes. Frequent human–bat interactions pose zoonotic risks. While no lyssavirus was detected in bats, ongoing surveillance is crucial. This first comprehensive genome analysis of Malawian RABVs highlights their regional transmission and signifies the need for regional collaboration in rabies control, community education, and further study of genetic adaptations.

Dataset: DOI number or link to the deposited dataset in cases where the dataset is published or set to be published separately. If the dataset is submitted and will be published as a supplement to this paper in the journal Data, this field will be filled by the editors of

Received: date
Revised: date
Accepted: date
Published: date

Citation: To be added by editorial staff during production.

Copyright: © 2025 by the authors. Submitted for possible open access publication under the terms and conditions of the Creative Commons Attribution (CC BY) license (http://creativecommons.org/licenses/by/4.0/).

the journal. In this case, please make sure to submit the dataset as a supplement when entering your manuscript into our manuscript editorial system.

Dataset License: license under which the dataset is made available (CC0, CC-BY, CC-BY-SA, CC-BY-NC, etc.)

Keywords: rabies virus; knowledge; practice; whole-genome sequencing; phylogenetic analysis; human–bat interaction; risk factors; Malawi

1. Summary (required)

This dataset contains a BLAST hit table of full-length lyssavirus genomes from Malawian cases (MW-labeled sequences) in dogs, humans, and cattle, compared against reference sequences in the NCBI database and closely related strains from South Africa, Zimbabwe, and Tanzania, providing insights into genetic diversity and regional transmission dynamics.

2. Data Description (required)

The columns include **Strain** (query sample identifier), **Description** (reference sequence details), **Query Cover** (percentage of the query covered), **Per. ident** (percentage identity), **Acc. Len** (reference sequence length), and **Accession** (NCBI reference sequence identifier) (Table 1).

Table S1. BLAST results for Malawian lyssavirus sequences

Strain	Query Cover	Per. ident	Acc. Len	Accession
MW_HUMAN_2019	100%	98.27	11923	MT454644.1
MW_HUMAN_2019	100%	98.25	11923	MT454644.1
MW_HUMAN_2019	100%	98.25	11875	KR906746.1
MW_HUMAN_2019	100%	98.24	11875	KR906746.1
MW_HUMAN_2019	100%	98.16	11915	KR906751.1
MW_HUMAN_2019	100%	98.16	11915	KR906751.1
MW_HUMAN_2019	100%	97.91	11801	KX148203.1
MW_HUMAN_2019	100%	97.89	11801	KX148203.1
MW_HUMAN_2019	100%	97.88	11890	KR906757.1
MW_HUMAN_2019	100%	97.85	11890	KR906757.1
MW_HUMAN_2019	100%	97.82	11923	KT336434.1
MW_HUMAN_2019	100%	97.82	11923	KT336434.1
MW_HUMAN_2019	100%	97.7	11922	KX148103.1
MW_HUMAN_2019	100%	97.65	11922	KX148103.1
MW_DOG_2019	100%	98.28	11923	KR906747.1
MW_DOG_2019	100%	98.28	11923	KR906747.1
MW_DOG_2019	100%	98.25	11923	MT454644.1
MW_DOG_2019	100%	98.24	11875	KR906746.1
MW_DOG_2019	100%	98.16	11915	KR906751.1

MW_DOG_2019	100%	97.89	11801	KX148203.1
MW_DOG_2019	100%	97.85	11890	KR906757.1
MW_DOG_2019	100%	97.82	11923	KT336434.1
MW_DOG_2019	100%	97.65	11922	KX148103.1
MW_DOG_2019	100%	97.36	11923	KT336435.1
MW_DOG_2019	100%	97.32	11800	KX148204.1
MW_DOG_2019	99%	97.26	11923	KT336433.1
MW_DOG_2019	100%	97.2	11918	MG458318.1
MW_DOG_2019	100%	97.2	11923	KT336432.1
MW_DOG_2019	100%	97.19	11923	MT454631.1
MW_DOG_2019	100%	97.17	11923	KT336436.1
MW_DOG_2019	100%	97.16	11923	MT454637.1
MW_DOG_2019	100%	97.16	11923	MT454634.1
MW_DOG_2019	100%	97.15	11923	MT454635.1
MW_DOG_2019	100%	97.15	11923	MT454652.1
MW_DOG_2019	100%	97.14	11923	MT454641.1
MW_DOG_2019	100%	97.14	11923	MT454639.1
MW_DOG_2019	100%	97.14	11923	MT454653.1
MW_DOG_2019	100%	97.14	11923	MT454648.1
MW_DOG_2019	100%	97.14	11923	MT454636.1
MW_DOG_2019	100%	97.13	11923	MT454654.1
MW_DOG_2019	100%	97.1	11923	KT336437.1
MW_DOG_2019	100%	97.08	11923	MT454642.1
MW_DOG_2019	100%	97.01	11923	JX473841.1
MW_DOG_2019	100%	96.92	11923	MT454638.1
MW_DOG_2019	100%	96.92	11923	JX473840.1
MW_DOG_2019	100%	96.88	11923	JX473839.1
MW_DOG_2019	100%	96.86	11923	JX473838.1
MW_DOG_2019	100%	96.79	11908	MT454633.1
MW_DOG_2019	100%	96.75	11801	KX148206.1
MW_DOG_2019	100%	96.74	11922	MT454645.1
MW_DOG_2019	100%	96.72	11923	MT454640.1
MW_DOG_2019	100%	96.72	11922	MT454651.1
MW_DOG_2019	100%	96.72	11922	MT454643.1
MW_DOG_2019	100%	96.71	11922	MT454650.1
MW_DOG_2019	100%	96.71	11922	MT454649.1
MW_DOG_2019	100%	96.71	11922	MT454646.1
MW_DOG_2019	100%	96.7	11922	MT454647.1
MW_DOG_2019	100%	96.64	11923	LT909534.1
MW_DOG_2019	100%	96.62	11906	KY210309.1
MW_DOG_2019	100%	96.62	11923	KR534252.2
MW_DOG_2019	100%	96.61	11920	KR906779.1

MW_DOG_2019	100%	96.59	11914	KR906744.1
MW_DOG_2019	100%	96.59	11801	KX148205.1
MW_DOG_2019	100%	96.59	11923	KR906739.1
MW_DOG_2019	100%	95.82	11914	KY210275.1
MW_COW_2021	100%	97.58	11801	KX148203.1
MW_COW_2021	100%	97.4	11923	KT336435.1
MW_COW_2021	99%	97.36	11923	KT336433.1
MW_COW_2021	100%	97.36	11923	KT336435.1
MW_COW_2021	100%	97.35	11922	KX148103.1
MW_COW_2021	100%	97.34	11800	KX148204.1
MW_COW_2021	100%	97.33	11800	KX148204.1
MW_COW_2021	100%	97.32	11800	KX148204.1
MW_COW_2021	99%	97.28	11923	KT336433.1
MW_COW_2021	100%	97.26	11923	KT336434.1
MW_COW_2021	99%	97.26	11923	KT336433.1
MW_COW_2021	100%	97.24	11801	KX148206.1
MW_COW_2021	100%	97.22	11918	MG458318.1
MW_COW_2021	100%	97.21	11923	KT336432.1
MW_COW_2021	100%	97.2	11918	MG458318.1
MW_COW_2021	100%	97.2	11923	KT336432.1
MW_COW_2021	100%	97.2	11923	MT454631.1
MW_COW_2021	100%	97.19	11923	MT454631.1
MW_COW_2021	100%	97.19	11923	KT336436.1
MW_COW_2021	100%	97.18	11923	MT454637.1
MW_COW_2021	100%	97.18	11923	MT454634.1
MW_COW_2021	100%	97.17	11923	KT336436.1
MW_COW_2021	100%	97.17	11923	MT454635.1
MW_COW_2021	100%	97.17	11923	MT454652.1
MW_COW_2021	100%	97.16	11914	KR906744.1
MW_COW_2021	100%	97.16	11923	LT909534.1
MW_COW_2021	100%	97.16	11923	MT454637.1
MW_COW_2021	100%	97.16	11923	MT454634.1
MW_COW_2021	100%	97.16	11923	MT454641.1
MW_COW_2021	100%	97.16	11923	MT454639.1
MW_COW_2021	100%	97.16	11923	MT454653.1
MW_COW_2021	100%	97.15	11801	KX148205.1
MW_COW_2021	100%	97.15	11923	MT454635.1
MW_COW_2021	100%	97.15	11923	MT454652.1
MW_COW_2021	100%	97.15	11923	MT454648.1
MW_COW_2021	100%	97.15	11923	MT454636.1
MW_COW_2021	100%	97.14	11923	KR534252.2
MW_COW_2021	100%	97.14	11923	MT454641.1

MW_COW_2021	100%	97.14	11923	MT454639.1
MW_COW_2021	100%	97.14	11923	MT454653.1
MW_COW_2021	100%	97.14	11923	MT454648.1
MW_COW_2021	100%	97.14	11923	MT454636.1
MW_COW_2021	100%	97.14	11923	MT454654.1
MW_COW_2021	100%	97.14	11923	KT336437.1
MW_COW_2021	100%	97.13	11923	MT454654.1
MW_COW_2021	100%	97.12	11923	KR906739.1
MW_COW_2021	100%	97.12	11906	KY210309.1
MW_COW_2021	100%	97.12	11921	KR906774.1
MW_COW_2021	100%	97.12	11869	KY210282.1
MW_COW_2021	100%	97.12	11923	KR534251.2
MW_COW_2021	100%	97.11	11920	KR906779.1
MW_COW_2021	100%	97.1	11923	KY210306.1
MW_COW_2021	100%	97.1	11923	KR906790.1
MW_COW_2021	100%	97.1	11923	KY210245.1
MW_COW_2021	100%	97.1	11923	KT336437.1
MW_COW_2021	100%	97.09	11923	KY210263.1
MW_COW_2021	100%	97.09	11923	KR906734.1
MW_COW_2021	100%	97.09	11914	KR906743.1
MW_COW_2021	100%	97.09	11919	KY210311.1
MW_COW_2021	100%	97.09	11923	KY210252.1
MW_COW_2021	100%	97.09	11923	KR534219.2
MW_COW_2021	100%	97.08	11914	KY210223.1
MW_COW_2021	100%	97.08	11923	MT454642.1
MW_COW_2021	100%	97.08	11923	MT454642.1
MW_COW_2021	100%	97.04	11884	KR534231.2
MW_COW_2021	100%	97.03	11923	KY210280.1
MW_COW_2021	100%	97.03	11914	KR534228.2
MW_COW_2021	100%	97.03	11923	KT336435.1
MW_COW_2021	100%	97.03	11923	KY210266.1
MW_COW_2021	100%	97.03	11923	MT454642.1
MW_COW_2021	100%	97.02	11923	KY210253.1
MW_COW_2021	100%	97.02	11922	KY210254.1
MW_COW_2021	100%	97.02	11922	KR906785.1
MW_COW_2021	100%	97.02	11923	KY210285.1
MW_COW_2021	100%	97.01	11923	KR534234.2
MW_COW_2021	100%	97.01	11867	KR534238.2
MW_COW_2021	100%	97.01	11923	JX473841.1
MW_COW_2021	100%	97.01	11923	JX473841.1
MW_COW_2021	100%	97	11885	KR534230.2
MW_COW_2021	100%	96.99	11923	LT909536.1

MW_COW_2021	100%	96.97	11923	MT454638.1
MW_COW_2021	100%	96.97	11923	KY210264.1
MW_COW_2021	100%	96.97	11923	JX473841.1
MW_COW_2021	100%	96.96	11923	KR906776.1
MW_COW_2021	100%	96.96	11920	KY210294.1
MW_COW_2021	100%	96.96	11914	KR906745.1
MW_COW_2021	100%	96.95	11920	KY210237.1
MW_COW_2021	100%	96.93	11923	KY210242.1
MW_COW_2021	100%	96.92	11923	MT454638.1
MW_COW_2021	100%	96.92	11923	JX473840.1
MW_COW_2021	100%	96.92	11923	MT454638.1
MW_COW_2021	100%	96.92	11923	JX473840.1
MW_COW_2021	100%	96.92	11923	JX473839.1
MW_COW_2021	100%	96.9	11923	JX473838.1
MW_COW_2021	100%	96.88	11923	JX473839.1
MW_COW_2021	100%	96.86	11923	JX473838.1
MW_COW_2021	100%	96.79	11908	MT454633.1
MW_COW_2021	100%	96.79	11801	KX148206.1
MW_COW_2021	100%	96.79	11908	MT454633.1
MW_COW_2021	100%	96.75	11801	KX148206.1
MW_COW_2021	100%	96.75	11922	MT454645.1
MW_COW_2021	100%	96.74	11922	MT454645.1
MW_COW_2021	100%	96.73	11922	MT454651.1
MW_COW_2021	100%	96.73	11922	MT454643.1
MW_COW_2021	100%	96.72	11923	MT454640.1
MW_COW_2021	100%	96.72	11922	MT454651.1
MW_COW_2021	100%	96.72	11922	MT454643.1
MW_COW_2021	100%	96.72	11922	MT454650.1
MW_COW_2021	100%	96.72	11922	MT454649.1
MW_COW_2021	100%	96.72	11922	MT454646.1
MW_COW_2021	100%	96.71	11922	MT454650.1
MW_COW_2021	100%	96.71	11922	MT454649.1
MW_COW_2021	100%	96.71	11922	MT454646.1
MW_COW_2021	100%	96.7	11922	MT454647.1
MW_COW_2021	100%	96.7	11923	MT454640.1
MW_COW_2021	100%	96.7	11922	MT454647.1
MW_COW_2021	100%	96.66	11923	LT909534.1
MW_COW_2021	100%	96.64	11923	LT909534.1
MW_COW_2021	100%	96.62	11906	KY210309.1
MW_COW_2021	100%	96.62	11923	KR534252.2
MW_COW_2021	100%	96.61	11920	KR906779.1
MW_COW_2021	100%	96.61	11906	KY210309.1

MW_COW_2021	100%	96.61	11923	KR534252.2
MW_COW_2021	100%	96.6	11923	KR534219.2
MW_COW_2021	100%	96.6	11869	KY210282.1
MW_COW_2021	100%	96.6	11923	KR534251.2
MW_COW_2021	100%	96.59	11914	KR906744.1
MW_COW_2021	100%	96.59	11801	KX148205.1
MW_COW_2021	100%	96.59	11923	KR906739.1
MW_COW_2021	100%	96.59	11923	KY210306.1
MW_COW_2021	100%	96.55	11854	KR906775.1
MW_COW_2021	100%	96.36	11914	KY210275.1
MW_COW_2021	100%	95.86	11914	KY210275.1
MW_COW_2021	100%	95.82	11914	KY210275.1

3. Methods (required)

Lyssavirus samples were collected from dogs, humans, and cattle in Malawi as part of rabies surveillance efforts. RNA was extracted, and full-length genomic sequences were obtained using the Illumina MiSeq platform.

Sequences were analysed using BLAST to compare them against the NCBI database, retrieving strain identity, query coverage, percentage identity, and reference accession details. Data were curated to remove duplicates and low-quality hits.

This study followed the Declaration of Helsinki (1975, revised 2008). Appropriate permits for wildlife sampling and analysis were obtained from the Department of National Parks and Wildlife, Malawi (Ref. No. DNPW 10 October 2014). Informed consent was obtained from all participants involved in the study.

Author Contributions: W.M. conceptualised the study, W.M., E.C., and H.K. collected the samples, N.S. and W.M. performed the laboratory and data analysis, H.S. provided financial support, N.S. prepared the original draft, and W.M., H.S., H.K., E.C., J.N., G.N., J.C., and R.T. reviewed and edited the manuscript. All authors have read and agreed to the published version of the manuscript.

Funding: This study was funded by grants from the Ministry of Education, Culture, Sports, Science and Technology, Japan (MEXT); the Japan Program for Infectious Diseases Research and Infra-structure (JIDRI) from the Japan Agency for Medical Research and Development (AMED) (JP23wm0125008); AMED (JP23fa627005); and Japan International Cooperation Agency (JICA) within the framework of the Science and Technology Research Partnership for Sustainable Development (SATREPS) (JP22jm0110019).

Institutional Review Board Statement: Appropriate permits for hunting, sampling wildlife, and analysis of both archived and collected samples in this research were obtained from the Department of National Parks and Wildlife, Malawi (Ref. No DNPW 10 October 2014).

Informed Consent Statement: Informed consent was obtained from all participants involved in the study.

Data Availability Statement: The datasets used and/or analysed during the current study are available from the corresponding author upon reasonable request.

Acknowledgments: We thank the technical staff of the University of Zambia, School of Veterinary Medicine, the Hokudai Center for Zoonosis Control in Zambia (HUCZCZ), Lilongwe University of Agriculture and Natural Resources (LUANAR), the Central Veterinary Laboratory and the Department of Animal Health and Livestock Development in Malawi for their invaluable support and contributions to this study.

Conflicts of Interest: The authors declare no conflicts of interest.

Abbreviations

The following abbreviations are used in this manuscript:

MDPI	Multidisciplinary Digital Publishing Institute
DOAJ	Directory of open access journals
TLA	Three letter acronym
LD	Linear dichroism

Disclaimer/Publisher's Note: The statements, opinions and data contained in all publications are solely those of the individual author(s) and contributor(s) and not of MDPI and/or the editor(s). MDPI and/or the editor(s) disclaim responsibility for any injury to people or property resulting from any ideas, methods, instructions or products referred to in the content.
